# *Clostridium difficile* Enteritis after Total Abdominal Colectomy for Ulcerative Colitis

**DOI:** 10.1155/2019/2987682

**Published:** 2019-02-03

**Authors:** Hassan Nasser, Semeret Munie, Dania Shakaroun, Tommy Ivanics, Surya Nalamati, Keith Killu

**Affiliations:** ^1^Department of Surgery, Henry Ford Hospital, 2799 W Grand Blvd, Detroit, MI 48202, USA; ^2^Department of Surgery/Division of Trauma and Critical Care, Henry Ford Hospital, 2799 W Grand Blvd, Detroit, MI 48202, USA; ^3^Department of Internal Medicine, Henry Ford Hospital, 2799 W Grand Blvd, Detroit, MI 48202, USA; ^4^Department of Surgery/Division of Colon and Rectal Surgery, Henry Ford Hospital, 2799 W Grand Blvd, Detroit, MI 48202, USA

## Abstract

**Introduction:**

Isolated* Clostridium difficile *small bowel enteritis is a rare condition with significant morbidity and mortality.

**Presentation of Case:**

An 83-year-old female with refractory ulcerative colitis underwent a total proctocolectomy and end ileostomy. Her postoperative course was complicated with return to the operating room for repair of an incarcerated port site hernia. Subsequently, she developed septic shock and multiorgan failure requiring intubation and mechanical ventilation, renal replacement therapy, and high dose vasopressors. Diagnostic workup revealed diffuse small bowel wall thickening on computed tomography scan as well as positive nucleic acid amplification test for* C. difficile *toxin B gene. Despite treatment with antibiotics and maximum attempts at resuscitation, the patient expired.* Discussion. C. difficile* infection most commonly affects the colon but rarely can involve the small bowel. The pathogenesis of* C. difficile *enteritis is unclear but is believed to mirror that of colitis. Surgical patients are susceptible for* C. difficile *infection, as they tend to be relatively immunosuppressed in the postoperative period. Radiologic findings of enteritis may mimic those of colitis and this includes small bowel dilation and thickening. Treatment for this condition has not been well established but it is approached similar to colitis.

**Conclusion:**

Despite an increase in the number of case reports of* C. difficile* enteritis, it continues to be a rare but potentially fatal infection. Clinicians should maintain a high index of suspicion especially in patients with inflammatory bowel disease who undergo colon resections.

## 1. Introduction

*Clostridium difficile *(*C. difficile*) is a gram-positive anaerobic spore-forming bacillus that is a major cause of nosocomial colitis. This infection is associated with antibiotic-induced changes to the normal intestinal microbial flora. The incidence of* C. difficile *infection has increased since the 2000s likely due to the rise in virulent strains and change in the pattern of antibiotic use [1]. Isolated* C. difficile *small bowel enteritis is a rare condition and up till 2013 there were only 83 cases described in the literature with a reported mortality of 23% [2]. We report a detrimental case of* C. difficile* enteritis in a patient with ulcerative colitis (UC) after a total proctocolectomy and end ileostomy.

## 2. Presentation of Case

**Figure 1:**
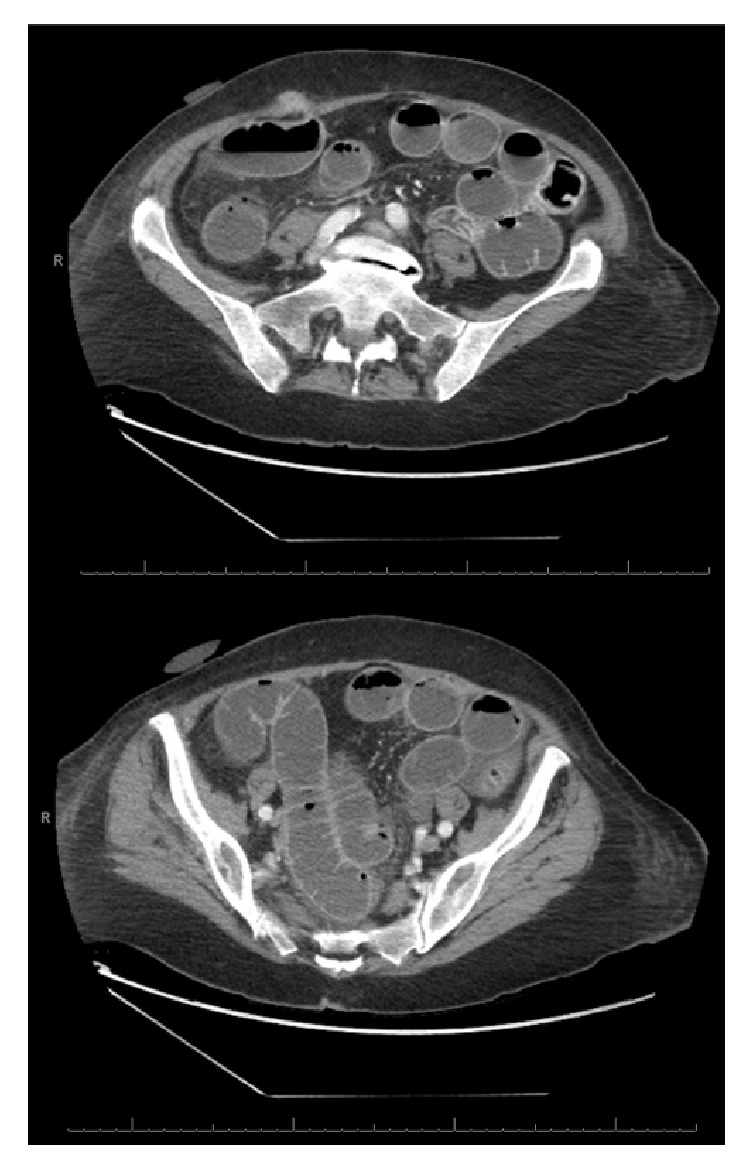
First CT scan abdomen and pelvis. Contrast-enhanced axial images showing port site hernia with diffuse small bowel dilation.

An 83-year-old female presented to the colon and rectal surgery clinic with a five-year history of UC resistant to medical management including 5-aminosalicylic acid, infliximab, adalimumab, and vedolizumab. She was maintained on low dose prednisone 5 mg daily. Her past medical history was significant for asthma and hypertension.* C. difficile *toxin and stool culture were checked a few months prior to her surgery and were negative. She was admitted to the hospital for an elective robotic-assisted laparoscopic total proctocolectomy and end ileostomy. No complications were noted intraoperatively. The patient received cefoxitin 2 grams for four doses preoperatively and intraoperatively (redosing every 3 hours). Pathology revealed chronic active pancolitis with no evidence of dysplasia. Postoperatively, the patient developed prolonged ileus with poor tolerance of oral intake and poor ileostomy function requiring insertion of a nasogastric tube and total parenteral nutrition. On postoperative day 7, the patient developed significantly worsening abdominal pain and distension. A computed tomography (CT) scan of the abdomen and pelvis was obtained revealing a right lower quadrant port site hernia with incarcerated small bowel obstruction (Figure 1). The patient was taken to the operating room for a diagnostic laparoscopy and repair of port site hernia. The bowel was viable and no bowel was resected. Two grams of cefazolin was given for perioperative prophylaxis.

She was admitted to the surgical intensive care for postoperative monitoring. Her condition began to deteriorate quickly on postoperative day 1 with worsening abdominal pain, abdominal distention, and increased ileostomy output of about 2000 mL per day. She developed kidney failure and respiratory failure necessitating intubation and mechanical ventilation. The patient developed septic shock and received a total of 8 liters of crystalloids and colloids with high doses of vasopressors. To maintain adequate mean arterial pressure and tissue perfusion, she required norepinephrine at a rate of 140 micrograms per minute, epinephrine at a rate of 13 micrograms per minute, and physiologic vasopressin at a rate of 0.04 units per minute. Intravenous hydrocortisone was also started for possible relative adrenal insufficiency. The patient developed severe refractory metabolic acidosis requiring bicarbonate infusion and renal replacement therapy. The patient was started on intravenous vancomycin, piperacillin-tazobactam, and fluconazole for possible sepsis.

**Figure 2:**
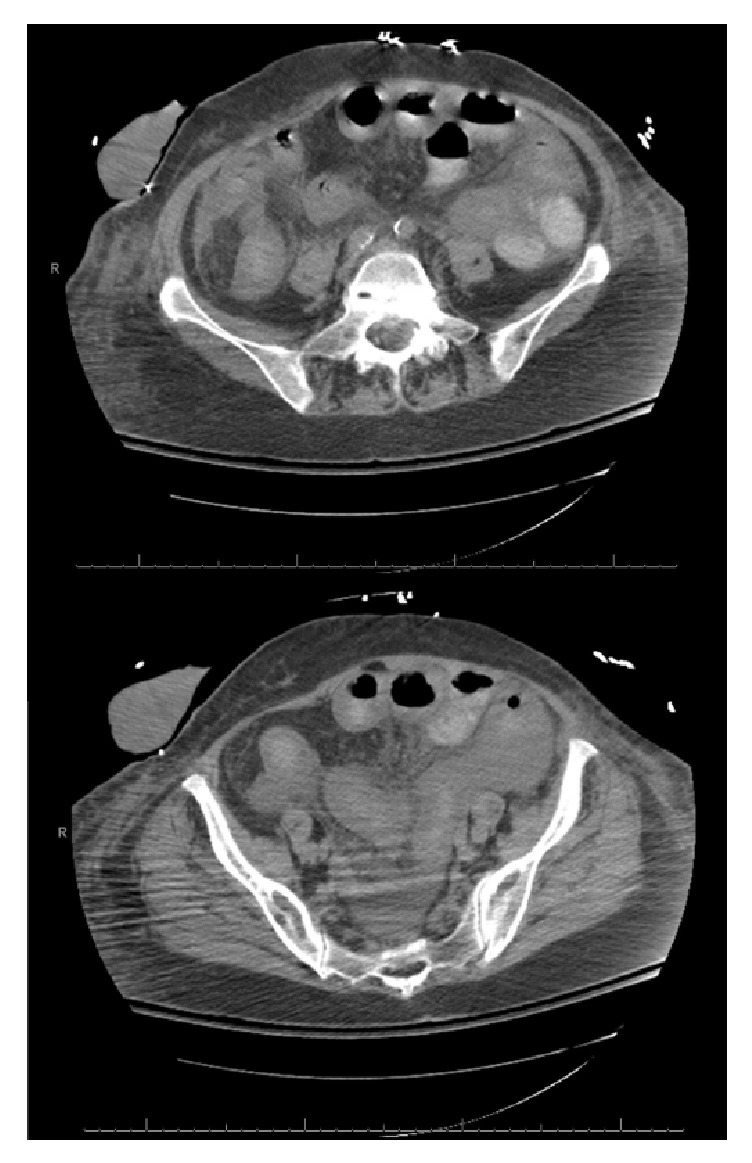
Second CT scan abdomen and pelvis two days later. Nonenhanced axial images showing diffuse small bowel dilation and intraperitoneal free fluid.

On postoperative day 2, laboratory studies showed a white blood cell count of 90,900 cells/mm^3^, hemoglobin 13.9 g/dL, platelets 145,000 cells/mm^3^, creatinine 2.5 mg/dL, sodium 133 mmol/L, chloride 110 mmol/L, albumin 1.1 g/dL, lactate 9.8 mmol/L, pH 7.13, arterial PCO_2_ 24.2 mmHg, HCO_3_ 7.7 mmol/L, and base deficit 20 mmol/L. Urine and blood culture did not grow any organisms. Chest X-ray showed no evidence of pneumonia. Abdominal X-ray showed centrally distended small bowel loops but was otherwise unremarkable. A repeat CT scan of the abdomen and pelvis was obtained showing diffuse small bowel dilation, bowel wall thickening, and free fluid in the abdomen (Figure 2).* C. difficile *toxins immunoassay was negative, but the nucleic acid amplification test confirmed the presence of toxin B gene. At this point, the patient was started on intravenous metronidazole and vancomycin through the nasogastric tube.

On postoperative day 3, despite maximal attempts to resuscitate the patient, she continued to require escalating and high doses of vasopressors, renal replacement therapy, and ventilatory support with no improvement in the shock state or the tissue perfusion. Fecal transplantation was being considered; however, the family decided to withdraw care at this point. The patient was made comfort care and expired shortly after.

## 3. Discussion

*C. difficile *is a common cause of nosocomial infections and is a major source of morbidity and mortality in the United States with an annual economic burden up to 3 billion dollars [3]. This infection most commonly affects the colon but rarely can involve the small bowel. Risk factors for development of* C. difficile *infection include recent antibiotic use, abdominal surgeries, immunosuppressants, gastric acid suppression, and inflammatory bowel disease (IBD) [3]. The pathogenesis of* C. difficile *enteritis is unclear but is believed to mirror that of colitis [4]. The development of* C. difficile *enteritis involves an alteration in the intestinal microbial flora which is most commonly caused by antibiotic usage. The most common antibiotics implicated in causing* C. difficile* infections are clindamycin, cephalosporins, and fluoroquinolones [3]. Our patient had abdominal surgery and received preoperative cefoxitin and cefazolin. She was also on low dose steroids and other immunosuppressive medications which may have further increased her risk of developing* C. difficile *enteritis. Surgical patients, especially colorectal patients and transplant recipients, are susceptible for* C. difficile *infection, as they tend to be relatively immunosuppressed in the postoperative period [3]. Most case reports of small bowel enteritis are reported in patient with history of IBD and after colon resection [5]. It has been reported that after the creation of an ileostomy, changes in the terminal ileal epithelium create an environment similar to that of the colon [6]. Kralovich et al. hypothesized that the function of the ileocecal valve with small bowel peristalsis prevents the colonization of the small bowel by* C. difficile *[7]. Both reasons possibly explain why patients are likely to develop* C. difficile* ileitis after a total colectomy and end ileostomy.

Patients with IBD commonly experience exacerbations secondary to* C. difficile *infection [8, 9]. Diagnosis and management of this infection in patients with IBD is quite challenging and, thus, it is important to test for* C. difficile *infection when patients present with a new acute IBD flare [8, 9]. In UC patients,* C. difficile *infection is associated with poorer outcomes [8]. Our patient was tested several times for* C. difficile *toxin and culture which were negative a few months prior to surgery. She had no new IBD flares or change in her health status in the interim. Therefore, repeat testing was not done immediately preoperatively.

*C. difficile* enteritis clinically presents similar to colitis with abdominal pain and tenderness, fever, high ileostomy or stool output, and leukocytosis [10]. The range of presentation could vary from mild to severe infections in the form of septic shock. As this pathology is rare, intensive care physicians should have a high clinical suspicion to make a prompt diagnosis and start empiric treatment. The diagnosis is confirmed with a molecular testing for toxins A and B. The enzyme immunoassays for toxins A and B are quick and simple to perform. They are associated with a low sensitivity; therefore, a false negative is common which was the case in our patient. Thus, a negative result is usually confirmed with the nucleic acid amplification test which is highly sensitive and specific [3]. An abdominal CT scan can show some findings consistent with* C. difficile* colitis which include colonic wall thickening, dilation, and ascites. However, there has been little literature on the findings of* C. difficile* enteritis on abdominal CT scan. There was a notable change in the patient's CT scan findings over the course of 2 days. The repeat CT scan revealed small bowel dilation, thickening, and moderate ascites. Siddiqui et al. describes a case report of* C. difficile* enteritis with similar abdominal CT scan findings [11]. Thus, this suggests that the radiologic findings of* C. difficile* enteritis may mimic those of colitis.

Since* C. difficile* enteritis is a rare infection, treatment has not been well established [4]. However, current treatment is similar to the treatment of colitis with enteral vancomycin or fidaxomicin as first line [12]. Surgical resection is considered if the infection is not controlled. The new recommendations were not in effect at the time of our patient encounter and, thus, the patient was treated with enteral vancomycin and intravenous metronidazole as per the previous guidelines. Nevertheless, the patient failed to improve. She was not a suitable candidate for surgical resection as her abdominal CT scan showed diffuse involvement of the small bowel with no evidence of compromise of her small bowel viability. As a last resort we considered fecal transplantation although there is no evidence of its use in small bowel* C. difficile *infection.

## 4. Conclusion

Despite an increase in the number of case reports of* C. difficile* enteritis, it continues to be a rare but potentially fatal infection. These patients will often be admitted to the intensive care unit for management of their sepsis. Clinicians should maintain a high index of suspicion especially in patients with IBD who undergo colon resections. It is still unclear what makes this patient population particularly susceptible to* C. difficile* enteritis. The presentation is usually severe and has the potential to progress to fulminant infection rapidly. Thus, early initiation of the appropriate treatment is crucial although more evidence may be required to identify the optimal therapy for this infection.

## Abbreviations

*Clostridium difficile*: * C. difficile*
Ulcerative colitis: UC
Computed tomography: CT
Inflammatory bowel disease: IBD.
